# Pectin-Based Formulations for Controlled Release of an Ellagic Acid Salt with High Solubility Profile in Physiological Media

**DOI:** 10.3390/molecules26020433

**Published:** 2021-01-15

**Authors:** Marco Aldo Ortenzi, Stefano Antenucci, Stefania Marzorati, Lucia Panzella, Silvia Molino, José Ángel Rufián-Henares, Alessandra Napolitano, Luisella Verotta

**Affiliations:** 1 Laboratory of Materials and Polymers (LaMPo), Dipartimento di Chimica, via Golgi 19, University of Milan, 20133 Milano, Italy; marco.ortenzi@unimi.it (M.A.O.); stefano.antenucci@unimi.it (S.A.); 2 Department of Environmental Science and Policy, via Celoria 2, University of Milan, 20133 Milano, Italy; stefania.marzorati@unimi.it; 3 Department of Chemical Sciences, University of Naples “Federico II”, via Cintia 4, 80126 Naples, Italy; panzella@unina.it (L.P.); alesnapo@unina.it (A.N.); 4 Departamento de Nutrición y Bromatología, Instituto de Nutrición y Tecnología de Alimentos, Centro de Investigación Biomédica, Universidad de Granada, 18071 Granada, Spain; silviamolino@correo.ugr.es (S.M.); jarufian@ugr.es (J.Á.R.-H.); 5 Instituto de Investigación Biosanitaria (ibs. GRANADA), Universidad de Granada, 18071 Granada, Spain

**Keywords:** ellagic acid lysine salt, pectin-based formulations, controlled release, gastrointestinal digestion, gut microbiota

## Abstract

Among bioactive phytochemicals, ellagic acid (EA) is one of the most controversial because its high antioxidant and cancer-preventing effects are strongly inhibited by low gastrointestinal absorption and rapid excretion. Strategies toward an increase of solubility in water and bioavailability, while preserving its structural integrity and warranting its controlled release at the physiological targets, are therefore largely pursued. In this work, EA lysine salt at 1:4 molar ratio (EALYS), exhibiting a more than 400 times increase of water solubility with respect to literature reports, was incorporated at 10% in low methoxylated (LM) and high methoxylated (HM) pectin films. The release of EA in PBS at pH 7.4 from both film preparations was comparable and reached 15% of the loaded compound over 2 h. Under simulated gastric conditions, release of EA from HM and LM pectin films was minimal at gastric pH, whereas higher concentrations—up to 300 μM, corresponding to ca. 50% of the overall content—were obtained in the case of the HM pectin film after 2 h incubation at the slightly alkaline pH of small intestine environment, with the enzyme and bile salt components enhancing the release. EALYS pectin films showed a good prebiotic activity as evaluated by determination of short chain fatty acids (SCFAs) levels following microbial fermentation, with a low but significant increase of the effects produced by the pectins themselves. Overall, these results highlight pectin films loaded with EALYS salt as a promising formulation to improve administration and controlled release of the compound.

## 1. Introduction

Ellagic acid (EA)—a polyphenol widely distributed in fruits, particularly in berries, nuts, and pomegranates—is presently the focus of high interest in the pharmacological and nutraceutical sectors because of its many biological activities. In addition to its remarkable antioxidant activity, its antiviral, antimicrobic [1], antiteratogenic [2], stimulation of type I collagen production and elastin protection in skin [3], and, most importantly, antitumor activities have been reported [4]. The latter results from an antiproliferative action combined with the ability to exert a protective function toward DNA against carcinogenic agents by adduct formation [5,6]. Chemopreventive and anti-inflammatory activities have been reported for urolithins derived by catabolism of EA by gut microbiota [7,8,9]. Other metabolites like methyl and dimethyl ethers and glucuronates are generated by EA following adsorption by ileum cells and have been found in the plasma, urine, and bile of animals fed with ellagitannins [9,10]. However, EA accumulates mainly in the intestinal epithelium, but only to a limited extent in the liver, lungs and blood [9].

The low aqueous solubility of EA and permeability in the gastrointestinal tract (GIT), its permanent binding to DNA and proteins of cells, and first pass metabolism are considered the main reasons for its low oral bioavailability and consequently its low therapeutic potential. In addition to low absorption in the GIT, EA is also rapidly eliminated, typically within 2–6 h, depending on food composition [6]. In a previous work, González-Sarrías et al. studied in vivo–in vitro human EA bioavailability, resulting in a large interindividual variability, identifying multiple key factors (low solubility of free EA under gastric conditions, type of precursor, limited intestinal EA absorption). In addition, even upon high free EA intake, EA concentration in plasma was always quite low [11]. It has been demonstrated that in the grand intestine tract, EA undergoes an extensive metabolism by the gut microbiota providing urolithins, which are 25–80-fold more bioavailable and much better absorbed than EA [12,13]. Based on these data, interest has significantly increased in recent years for the development of delivery systems that might increase the solubility in water and bioavailability of EA, preserve its structural integrity, and warrant its controlled release at the physiological target [14].

In this frame, in a recent study we have shown that EA is a main constituent (40% on a *w*/*w* basis) of the alcoholic extracts of the wastes deriving from production of wines by yeast fermentation of *Punica granatum* [15]; the fermented residues are able to slowly release EA up to 80% of the overall content over 2 h incubation at the slightly alkaline pH, simulating the small intestine environment. Nanosponges based on cyclodextrin and cross-linked by dimethyl carbonate loaded with EA have been reported to improve its solubilization and oral bioavailability in animal models [16]. Schizophyllan–chitin-based nanoparticles have been proposed to encapsulate EA as an anticancer agent, improving its effects [17]. However, the possibility to exploit biocompatible or natural biopolymers that are readily available and commonly used as oral drug delivery systems for improving EA solubility and to increase its bioavailability in the gut, minimizing its release in the upper GIT, has so far been scarcely explored. To this aim of particular interest are pectins, a soluble dietary fiber—containing mainly partially methoxylated galacturonic acid—that is available at low cost. Pectins are widely used in food industries (food additive E440 according to International Numbering System for Food Additives (INS)) as gelling or thickening agents and in the pharmaceutical industry as bulking agents in drug production, in wound healing preparations, and in medical adhesives for oral drug delivery formulations (e.g., controlled release systems, gastro-retentive systems, colon-specific delivery systems, and mucoadhesive delivery systems). Beads prepared from sodium alginate and pectin with dual cross-linking were tested for effective control of release of the antidiabetic drug repaglinide [18]. Numerous applications have been reported that exploit the colon-specific delivery ability of pectins. These include: (a) the chitosan/layered double hydroxide biohybrid beads coated with pectin that proved efficient for controlled release of 5-aminosalycilic acid in the treatment of colon diseases [19]; (b) gel microspheres by ionotropic gelation synthesized from pectins with different methylation degrees for encapsulation of sulfasalazine, a nonsteroidal anti-inflammatory drug used mainly for the treatment of an inflammatory bowel and other diseases [20]; and (c) calcium pectinate gel beads for the release of prednisolone [21]. Moreover, due to the presence of highly polar functional groups, pectins have been used for the controlled release of polyphenols. Citrus pectin encapsulation of anthocyanins was found to increase intestinal accessibility during passage through the small intestine in healthy volunteers [22].

In the present study, high methoxylated pectin (HM) extracted from apple and low methoxylated pectin (LM) extracted from citrus peel were investigated for the controlled release of EA. A salt of EA with lysine (EALYS) was prepared to improve water solubility and loading into the pectin matrix. The release of EA from these formulations under simulated gastrointestinal conditions was evaluated as well as the prebiotic activity on the gut microbiota.

## 2. Results and Discussion

### 2.1. EALYS Preparation

**Figure 1:**
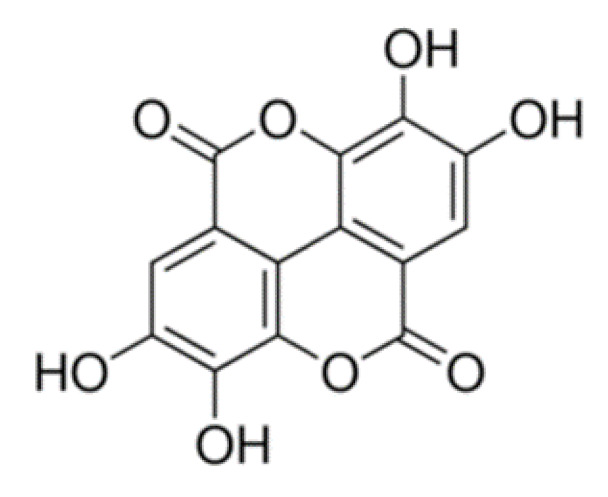
Ellagic acid (EA).

EA (structure reported in Figure 1) was solubilized in water in the presence of variable amounts of lysine. The results show that only EALYS 1:4 salt is soluble in water (up to 13 mg/mL). Its ^1^H-NMR spectrum confirmed protonation of the amino group in lysine and the presence of EA signal at 7.13 ppm and those from lysine at higher fields. According to literature, the water solubility of EA is 9.7 μg/mL [23]; therefore, considering that EA represents 34% *w*/*w* of EALYS, the water solubility of EA increased more than 400 times thanks to the salt formation.

**Figure 2:**
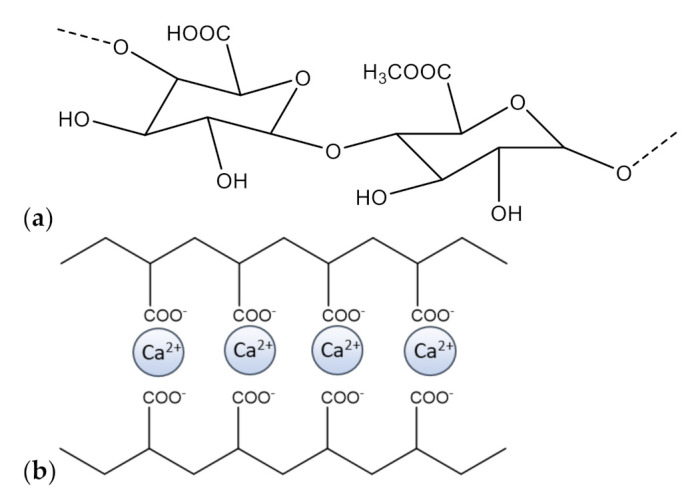
(**a**): pectin structure. (**b**): Schematic interaction between calcium ions and pectin COO^−^ groups.

Solubility studies were also conducted using a CaCl_2_ water solution, since the latter is used to obtain pectin gels, and in PBS solution (pH = 7.4), used for release tests to mimic physiological media. The solubility of EALYS dramatically decreased when calcium chloride was added to water, dropping to 0.31 mg/mL, i.e., less than 2.5% of the solubility of EALYS 1:4 in pure water. Examples of limited solubility of calcium salts of phenols were already reported [24]; therefore, the formation of a very low water-soluble calcium–EA salt could explain the reduction of solubility of EALYS in water solution. Nevertheless, Ca^2+^ is fundamental to obtaining pectin gels, as already described in previous literature [21], due to the ion-exchanging reaction occurring with –COOH pectin groups (Figure 2).

Additionally, PBS reduced the solubility of EALYS in comparison with water, leading to a solubility of 8.95 mg/mL. This can be explained considering that phosphate ions interfere with lysine and lead to EA partial precipitation.

In conclusion, in 1:4 EALYS, the excess of lysine permitted the solubilization of the salt acting as a compatibilizer between water and EA–lysine diprotic salt, allowing us to obtain for subsequent pectin film preparation a highly water-soluble form of EA that could be used with a final concentration of 0.31 mg/mL, more than 30 times higher than the solubility of EA alone in water. 

### 2.2. Optimization of Pectin Film Preparation

#### 2.2.1. Degree of Esterification Determination

**Figure 3:**
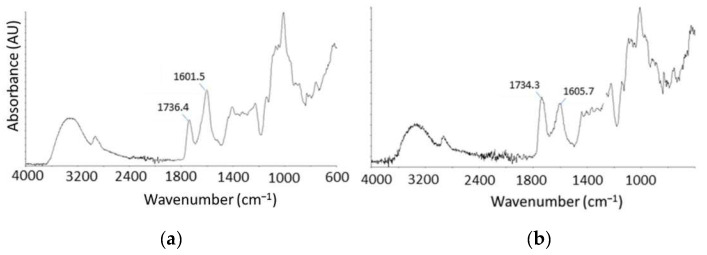
ATR-IR spectra of pectin samples. (**a**): pectin from citrus peel (LM). (**b**): pectin from apple (HM).

Two pectins at different degrees of esterification (DEs) were used for the preparation of films, namely low methoxylated pectin (LM) pectin (DE ≈ 50%) and high methoxylated pectin (HM) pectin (DE ≈ 70%). The exact DE to properly evaluate the amount of CaCl_2_ necessary to obtain the gels was calculated according to the procedure indicated in paragraph 3.6 by checking the bands at ≈1601 and ≈1740 cm^−1^ as reported in literature [20]. Spectra of LM and HM pectins are reported in Figure 3.

The band at ca. 1601 cm^−1^ is due to the symmetrical stretching vibration of carboxylate moiety, whereas the band at ca. 1740 cm^−1^ is assigned to stretching of carbonyl groups from both carboxylic acid and methyl ester moieties. Considering that at pH 6 in partially methoxylated pectin the carboxylic groups were completely deprotonated, the 1740 cm^−1^ band can be attributed exclusively to the carboxymethyl groups. The ratio between the absorbance intensity at 1740 cm^−1^ divided by the sum of those at 1601 and 1740 cm^−1^ is proportional to DE. The equation reported by Manrique et al. [25] that correlates the DE to absorbance parameters was used for DE determination of the pectins. According to this method, a degree of esterification of 59.3% was determined for citrus peel pectin (LM), whereas a degree of esterification of 70.9% (HM) was assessed for apple-based pectin.

#### 2.2.2. Rheological Analyses

**Figure 4:**
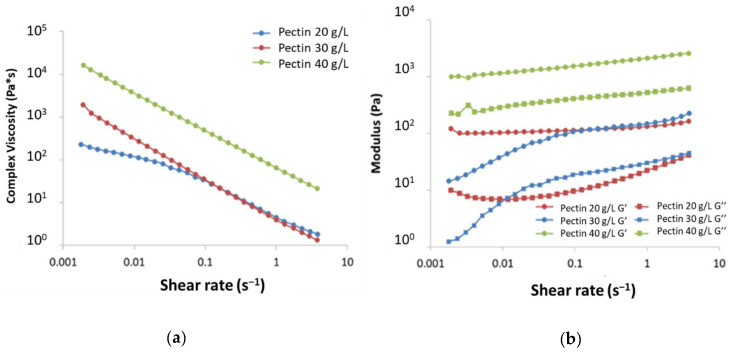
Rheological analysis of pectin gels. (**a**): complex viscosity (η*); (**b**): storage modulus (G’) and loss modulus (G”) vs. shear rate of 20 g/L, 30 g/L, and 40 g/L LM pectin gels.

Several pectin concentrations were tested to obtain gels: frequency sweep experiments showed that both for HM and for LM pectin, low concentrations, i.e., 3, 5, 7.5, and 10 g/L, led to gels having very low viscosity that were not sufficient to provide a stable and tough gel (data not shown). On the other side, pectin concentrations of 20 g/L and higher led to stable and tough gels having a storage modulus (G’) significantly higher than the loss modulus (G”), with the highest pectin concentration (40 g/L) resulting in a highly viscous gel (Figure 4). Given the results obtained, 30 g/L was chosen as the best pectin concentration for the development of EALYS-containing gels: tests were also repeated after 168 h, confirming that the gel was stable and no depolymerization of pectin occurred (see Figure S1 in Supplementary Materials).

Since the release of active principles can be affected by the viscosity of the gel, the rheology of EALYS-loaded gels was checked: the presence of EALYS did not affect the viscosity of the gels immediately after the gelling period (48 h). However, after a week, at low shear rate, the viscosity displayed an anomalous decreasing trend. This can be explained considering that, as discussed before, EALYS shows a decreased solubility in water when Ca^2+^ is present, due to the formation of EA(Ca)_n_(LYS)_m_ salts. Probably, the reaction of EA with calcium ions subtracted Ca^2+^ from the pectin network reducing the viscosity of gels. Although a decrease in viscosity for shear rate lower than 0.01 s^−1^ was registered, the G’ modulus was still higher than the G’’ modulus (see Figure S2 in Supplementary Materials); therefore, the solid structure of pectin gel was not yet compromised. This confirms that the gel retained the solid structure even when EALYS solubility was reduced due to the presence of calcium ions.

### 2.3. Release Experiments

**Figure 5:**
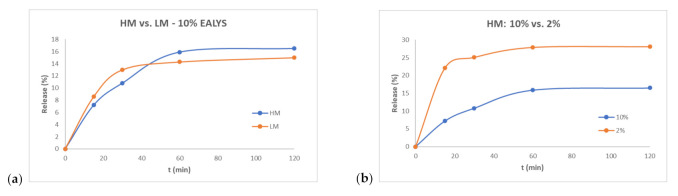
Release kinetics for the EA with lysine (EALYS)-loaded LM and HM pectin films in phosphate buffered saline (PBS) at pH 7.4 at 2 *w*/*w*%. (**a**): Release from LM + 10% EALYS vs. HM + 10% EALYS. (**b**): Release from HM + 10% EALYS vs. HM + 2% EALYS.

The release of EALYS from LM + 10%EALYS and HM + 10%EALYS films were compared in order to determine the effect of DE on the release properties. Both samples showed the same behavior: the quantity of EALYS released after 120 min was very similar, i.e., about 15% and 17% with LM and HM pectin gels, respectively, and in both cases a plateau was reached after 60 min (Figure 5). Additionally, HM + 2% EALYS release was assessed: a burst release was observed, since after only 20 min 25% of EALYS was released. Plateau was reached after about 60 min, when about 28% of EALYS was released. Although the relative quantity of EALYS released was higher in the 2% sample, the absolute quantity released was far higher in samples loaded with 10% EALYS; therefore, 2% samples were not further investigated. In any case, release after 24 h was not significantly different from that obtained after 120 min.

### 2.4. Release of EA from Pectin Films under Simulated Gastrointestinal Conditions

**Figure 6:**
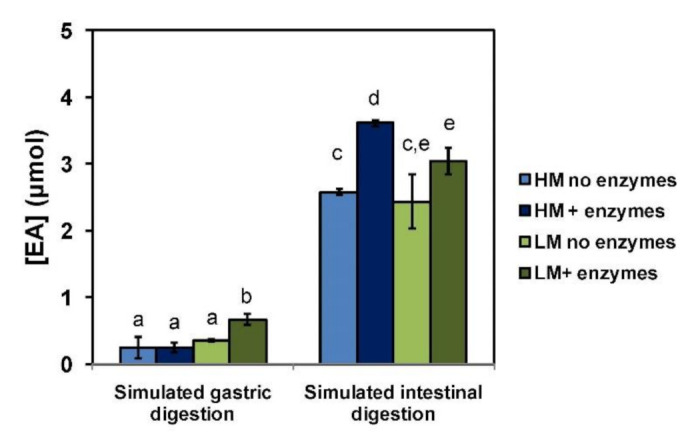
Concentration of EA released under simulated gastrointestinal digestion from pectin films. Reported are the mean ± SD values of three experiments. Different letters indicate statistically significant differences (*p* < 0.05).

To further explore the potential use of HM and LM pectin films for the controlled release of EA under physiological conditions, both materials were subjected to simulated gastrointestinal conditions following a reported protocol [15,26,27]. A limited amount of EA, ranging from ca. 0.2 to 0.7 μmol (25–66 μM concentration, corresponding to 4–12% of the overall EA content), was released after the first incubation at gastric pH, whereas higher quantities, up to 3.6 μmol in the case of the HM pectin film (ca. 300 μM concentration, corresponding to 64% of the overall content), were measured after 2 h incubation at the slightly alkaline pH, simulating the small intestine environment (Figure 6). Notably, a less efficient release of EA, especially at intestinal pH, was observed in the absence of enzymes or bile salts. This would suggest that EA release is controlled not only by the different solubility properties of the compound at the acidic and neutral pH mimicking the stomach and intestinal environment, respectively, but also by interactions of the phenol with the pectin polymeric network. These latter were significantly affected by the digestive enzymes and bile salts, resulting in a weakening of the EALYS and pectin interactions and hence in a higher release of EA.

### 2.5. Prebiotic Activity of EALYS-Containing Pectin Films

It is well known that non-starch polysaccharides such as pectins may exert beneficial effects on human health, preventing the onset of inflammatory bowel disease and related pathologies by modulation of gut microbiota activity [28,29,30]. This generally results in an increase in the production of short chain fatty acids (SCFAs), whose healthy properties, e.g., immunomodulation, are well established [31]. The same has been reported for dietary phenolic compounds, including EA [32,33]. On this basis, the prebiotic activity of the EALYS pectin films was evaluated by determination of changes in production of SCFAs following microbial fermentation. Table 1 shows the levels of acetic, propionic, and butyric acids released after fermentation of the EALYS-loaded pectin films compared with those produced by the control films and a blank sample (fermentative medium only). As expected, fermentation of all the pectin films released higher amounts of SCFAs compared with the blank sample (except for neat LM pectin films in the case of acetic acid), but these were invariably higher when EALYS was present. Moreover, an increased prebiotic activity was observed for HM pectin films with respect to LM films, except for butyric acid production. These findings open the way toward a systematic evaluation of EA pectin films for application in human health, e.g., as food supplements.

## 3. Materials and Methods

### 3.1. Reagents

Two types of pectins were purchased from Sigma-Aldrich (Schnelldorf, Germany): pectin from apple (Poly-d-galacturonic acid methyl ester), having a degree of esterification between 50% and 75%; and pectin from citrus peel, having a degree of esterification higher than 67%. Calcium chloride anhydrous (≥99.9%, 40 mesh) and disodium hydrogen phosphate (anhydrous, ≥98.0%) were purchased from Merck (Darmstadt, Germany). Ellagic acid (≥96.0%) and l-lysine (crystallized, ≥98.0%) were purchased from Fluka (Buchs, Switzerland). Pepsin, pancreatin, bile salts, peptone water, and resazurin were purchased from Sigma-Aldrich (Schnelldorf, Germany). Distilled water served as the solvent for preparing film solutions.

### 3.2. Liquid Chromatography

HPLC analysis of EA during in vitro digestion was run on an Agilent 1100 Series instrument using a reverse phase Phenomenex Sphereclone 250 × 4.60 mm column (particle size 5 µm). A gradient elution at a flow rate of 0.7 mL/min was performed using 0.1% formic acid in water (eluant A) and methanol (eluant B): 5% B, 0–10 min; 5–80% B (10–45 min); detection wavelength was fixed at 254 nm.

Short chain fatty acids (SCFAs) determination was carried out on an Accela 600 HPLC (Thermo Scientific, Munich, Germany) equipped with a pump, an autosampler, and a UV–VIS PDA detector set at 210 nm; the mobile phase used was 0.1 M phosphate buffer (pH 2.8)/acetonitrile 99:1 *v*/*v* delivered at a 1 mL/min flow rate; the column used was an Aquasil C18 reversed phase (Thermo Scientific, Munich, Germany) (150 × 4.6 mm, 5 µm), with a total run time of 20 min.

### 3.3. Ellagic Acid–Lysine Salt (EALYS) Preparation

EA (0.498 g, 1.65 mmol) and l-lysine (molar ratio of 1:1, 1:2, 1:3, and 1:4 on EA) were weighted in a 100 mL round bottom flask and solubilized in 20 mL of distilled water under magnetic stirring at room temperature. After 30 min, water was removed under vacuum at 50 °C using a rotary evaporator until a wet powder was obtained. Residual water was then removed under high vacuum (2 × 10^−2^ kPa) for 10 h at room temperature.

Each EALYS salt (20 mg) was weighted in a 15 mL glass vials and aliquots of 0.1 mL of Chromasolv water was added under magnetic stirring at room temperature (20 °C) until a homogeneous solution was obtained. Solubility of the salts are shown in Table 2. In the case of EALYS 1:1, 1:2, and 1:3 salts, the salt was still visible after the addition of 15 mL of water; the sample was then centrifuged, and water was removed with a pipette. The wet salt was then dried under high vacuum (2 × 10^−2^ kPa) for 10 h at room temperature and weighted to check that the residual weight that resulted was almost identical to the initial one.

### 3.4. H-Nuclear Magnetic Resonance (^1^H-NMR)

The NMR spectrum of EALYS 1:4 was recorded using a Bruker 400 MHz NMR on 10 mg of EALYS 1:4 dissolved in 1 mL of deuterium oxide (D_2_O). ^1^H-NMR (400 MHz, D_2_O): δ 7.13 (1H, s), 3.58 (2H, t, *J* = 6 Hz), 2.90 (4H, t, *J* = 8 Hz), 1.75 (4H, m), 1.60 (4H, dd, *J* = 8, 6 Hz), 1.35 (4H, m).

### 3.5. Solubility in CaCl_2_ Water Solution and in Phosphate Buffer Solution (PBS)

The solubility of EALYS 1:4 in CaCl_2_ solution and in phosphate buffer saline (PBS) was estimated via UV analysis against a calibration curve built using EALYS water solutions, setting the wavelength at 280 nm. A Jasco V-630 spectrophotometer was used. EALYS (22.5 mg) was weighted in a 100 mL volumetric flask and solubilized in water. Twenty mL of this solution was transferred in a 100 mL volumetric flask, which was filled with Chromasolv water (Sol A). Sol A was then diluted to obtain a total of 5 solutions having different concentrations of EALYS 1:4 salt.

EALYS 1:4 (12 mg) and CaCl_2_ (8 mg) were weighted in a 100 mL conic flask and dissolved in 50 mL of water. The flask was taken in a water shaking bath for 60 min at 37 °C, then filtered using a syringe equipped with a 0.45 μm Teflon filter, and the resulting solution was analyzed. Samples were prepared in triplicate in order to validate the results.

EALYS 1:4 (200 mg) was weighted in a 100 mL conic flask and dissolved in 50 mL of PBS. The flask was taken in a water shaking bath for 60 min at 37 °C, then filtered using a syringe equipped with a 0.45 μm Teflon filter, and the resulting solution was analyzed. Samples were prepared in triplicate in order to validate the results.

### 3.6. Determination of Degree of Esterification (DE) of Pectins

The DE of pectins was determined according to the method developed by Manrique et al. [25] using a Spectrum 100 spectrophotometer (Perkin Elmer) in attenuated total reflection (ATR) mode using a resolution of 4.0 and 256 scans in a range of wavenumber between 4000 cm^−1^ and 400 cm^−1^. A single-bounce diamond crystal was used with an incidence angle of 45°.

Pectin was dissolved in water (concentration of 5 g/L) under magnetic stirring at room temperature overnight; then the pH was adjusted at 6.0 using 1 M KOH. Water was removed by freeze-drying in order to obtain solid powders that were then analyzed.

### 3.7. Pectin Gels Preparation and Casting

Pectin gels were prepared following the procedure described by Fu et al. [34] and Tibbits et al. [35] using CaCl_2_. For each pectin, several samples were prepared using concentrations of pectin of 3 g/L, 5 g/L, 7.5 g/L, 10 g/L, 20 g/L, 30 g/L, and 40 g/L. In brief, pectin was weighted in a 100 mL round bottom flask and dissolved in 0.1 M NaCl water solution under magnetic stirring overnight. The pH was then adjusted to between 6.5 and 7.0 by using 1 M KOH water solution and the solution was mechanically stirred at 80 °C for 15 min. Solid CaCl_2_ was added, keeping the solution under mechanical stirring for 10 min at 80 °C in order to obtain a homogenous solution. The solution was then left at room temperature for 48 h in order to complete the gelling process.

The amount of CaCl_2_ required for the gelling process was calculated based on the DE of each pectin using Equation (1):

(1) $$R=\frac{2\left[{\mathrm{Ca}}^{2+}\right]}{\left[{\mathrm{COO}}^{-}\right]}$$

where *R* = 0.58 is a predetermined parameter already used in the procedure described in literature [34], [COO^−^] is the concentration on carboxylic acid moieties (determined knowing the DE) and [Ca^2+^] is calcium ion concentration.

### 3.8. EALYS Pectin Gels Preparation and Casting

A procedure close to procedure described in 3.9 was used for the preparation of pectin gels with EALYS 1:4. LM or HM pectin was weighted in a 100 mL round bottom flask and dissolved in 0.1 M NaCl water solution (30 g/L final concentration) under magnetic stirring overnight. The pH was adjusted to between 6.5 and 7.0 by using 1 M KOH, and then EALYS 1:4 was added (10% *w*/*w* on the solution). The solution was mechanically stirred for 10 min, then the temperature was raised to 80 °C for 15 min. Solid CaCl_2_ was added, keeping the solution under mechanical stirring for 10 min at 80 °C in order to obtain a homogenous solution. When gelification was required, the solution was left at room temperature for 48 h in a closed flask in order to complete the gelling process.

Pectin films were directly obtained by solvent casting deposition without waiting the 48 h indicated above, pouring 50 mL of a 30 g/L solution of pectin into a plastic Petri dish having 8.5 cm of diameter and 1 cm of depth. The film was obtained by solvent evaporation after 72 h under suction hood.

### 3.9. Rheological Analyses

Rheological analyses, conducted using frequency sweep experiments, were performed with a Physica MCR 300 rotational rheometer with a parallel plate geometry (Φ = 50 mm, distance between plates = 1 mm). Linear viscoelastic regimes of neat pectin gel and pectin gel loaded with EALYS were studied; strain was set equal to 3% and curves of complex viscosity as a function of frequency were recorded, taking 30 points ranging from 0.01 Hz to 20 Hz with a logarithmic progression, at 25 °C; in this paper, shear rate but not angular frequency is reported on the *x*-axis.

### 3.10. Release Experiments

EALYS pectin films (10–30 mg) were weighed in a 15 mL flat bottom vial and dispersed in phosphate buffered solution (PBS) at pH 7.4 (10 mL). The release assay was developed in a time range of 24 h by analyzing the samples at 15, 30, 45, 60, 120 min, and 24 h. Each sample was prepared in triplicate. The vials were placed in a horizontal water shaking bath (Dubnoff) set at 37 °C with a shaking ratio of 90 strokes per minute. At the fixed time, three samples were removed from bath, hand-shaken in order to obtain a homogeneous solution, and filtered using a syringe equipped with a 0.45 μm Teflon filter. The filtered solutions were analyzed by using a UV-VIS spectrophotometer in order to evaluate the quantity of EALYS released from pectin film.

### 3.11. In Vitro Digestion

A previously reported procedure was adopted [15,26,27]. Briefly, pectin films (50 mg) containing 10% *w*/*w* EALYS 1:4 were added to 10 mL of distilled water preliminarily adjusted to pH 2 with 1 M HCl. Added was 315 U/mL pepsin, and the mixture was incubated at 37 °C under stirring in the dark. After 2 h, 1 mL of solution was withdrawn and analyzed for EA by HPLC. To the remaining mixture, 9 mg of pancreatin and 56 mg of bile salts dissolved in 2.2 mL of 0.1 M NaHCO_3_ were added, followed by 0.8 mL of 1 M NaHCO_3_. After 2 h of incubation at 37 °C under stirring in the dark, the solution was again analyzed by HPLC. In order to stop the reaction, the mixture was then centrifuged at 14,000 rpm for 10 min at 4 °C. After separating the solid from the liquid fraction, 10% of this latter (1.2 mL) was added to the solid fraction, and this combined material was lyophilized. The lyophilized sample, mimicking the fraction that is not readily absorbed after digestion, was then subjected to the in vitro fermentation as described in Section 3.12. Control experiments were run exposing pectin films to the same pH conditions but without the addition of the enzyme components and bile salts.

### 3.12. In Vitro Fermentation and SCFAs Analysis

A previously described protocol was used [36]. In brief, to 50 mg of lyophilized digestion solid residue, 3 mL of distilled water was added into a screw-cap tube. Then, 1.25 mL of fermentation final solution (peptone water + resazurine) was added, followed by 0.33 mL of inoculum, consisting of a 32% feces solution in 0.1 M phosphate buffer (pH 7.0) (fecal content composed of a mixture of equal weight of fresh morning feces of three healthy adult human donors). Nitrogen was bubbled in order to reach an anaerobic atmosphere, and the mixture was incubated at 37 °C for 20 h under oscillation. The samples were then buried in ice to stop microbial activity and centrifuged, filtered through a 0.22 µm nylon filter, and finally analyzed for SCFAs (acetic, propionic, and butyric acid) by HPLC. A calibration curve was used for quantitation. Control samples not containing pectin films were also analyzed.

## 4. Conclusions

The use of pectins, an easily accessible low-cost class of heteropolysaccharides, as matrices to deliver bioactive compounds represents a green approach that has been increasingly pursued in the biomedical and nutraceutical fields since these soluble non-starch polysaccaharides meet most regulations for use in humans.

The results of this study highlight the good performance of pectins at low and high esterification degrees as systems for the controlled release of EA. The possibility that EA may be selectively released and absorbed in the intestinal tract after passing the gastric compartments was preliminarily demonstrated by use of a simulated gastric intestinal digestion model, suggesting that oral administration of EA may be conceived using such formulations. Degradation of the pectin network by the intestine enzymatic systems as suggested by our data may further aid the release. The concentrations reached particularly for HM pectin films may be reasonably near to the doses needed for bioactivity. Furthermore, the slight but significant prebiotic activity shown by the EALYS pectin films contribute to define the beneficial profile of the formulations presented. Further assessment of their properties on in vitro cellular systems and in vivo models represents a highly desirable perspective opened by this proof of concept study.

## Figures and Tables

**Table 1:** Prebiotic activity of pectin films measured as release of short chain fatty acids (SCFAs) (mmol/g of sample) ^1,2^.

Sample	Acetic Acid	Propionic Acid	Butyric Acid
HM Pectin + 10% EALYS	4.37 ± 0.09 ^a^	3.01 ± 0.06 ^a^	0.300 ± 0.007 ^a^
HM Pectin	3.89 ± 0.08 ^b^	2.06 ± 0.05 ^b^	0.220 ± 0.006 ^b^
LM Pectin + 10% EALYS	4.0 ± 0.1 ^b^	2.83 ± 0.09 ^c^	0.44 ± 0.02 ^c^
LM Pectin	2.89 ± 0.06 ^c^	1.73 ± 0.03 ^d^	0.280 ± 0.009 ^d^
Blank	1.01 ± 0.02 ^d^	0.94 ± 0.02 ^e^	-

^1^ Reported are the mean ± SD values of three experiments. ^2^ Different letters indicate statistically significant differences (*p* < 0.05).

**Table 2:** Water solubility of EALYS salts.

EALYS Salt	Weight (mg)	Water (mL)	Water Solubility (mg/mL)	CaCl_2_ Water Solution Solubility (mg/mL)	PBS Solubility (mg/mL)
1:1	20.1	15	Insoluble	------	------
1:2	23.6	15	Insoluble	------	------
1:3	19.8	15	Insoluble	------	------
1:4	22.4	1.7	12.99	0.31	8.95

## Data Availability

Data contained within the article or supplementary materials are available from the authors.

## Supplementary Materials

The following are available online, Figure S1: Rheological analysis of 30 g/L LM pectin gels after 48 h and after 168 h. Figure S2: Rheological analysis of 30 g/L LM pectin gels loaded with EALYS after 48 h and after 168 h. Figure S3: Rheological analysis of 40 g/L HM pectin gels and of 40 g/L HM pectin gels loaded with EALYS.

## Sample Availability

Samples of pectin gels, EALYS are available from the authors.
