# Supplementary material for: Pectin-Based Formulations for Controlled Release of an Ellagic Acid Salt with High Solubility Profile in Physiological Media
Source: Molecules. 2021 Jan 15;26(2):433. doi: 10.3390/molecules26020433 (PMC7829853; doi:10.3390/molecules26020433)
Supplement: Supplementary file 1 [file molecules-26-00433-s001.pdf]

molecules

Article

Pectin-Based Formulations for Controlled Release of an Ellagic Acid Salt with High Solubility Profile in Physiological Media

Marco Aldo Ortenzi [1], Stefano Antenucci [1], Stefania Marzorati [2], Lucia Panzella [3], Silvia Molino [4], José Ángel Rufián-Henares [4,5], Alessandra Napolitano [3], and Luisella Verotta [2,*]

[1] Laboratory of Materials and Polymers (LaMPo), Dipartimento di Chimica, via Golgi 19, University of Milan, 20133 Milano, Italy; marco.ortenzi@unimi.it (M.A.O.); stefano.antenucci@unimi.it (S.A.)
[2] Department of Environmental Science and Policy, via Celoria 2, University of Milan, 20133 Milano, Italy; stefania.marzorati@unimi.it
[3] Department of Chemical Sciences, University of Naples "Federico II", via Cintia 4, I-80126 Naples, Italy; panzella@unina.it (L.P.); alesnapo@unina.it (A.N.)
[4] Departamento de Nutrición y Bromatología, Instituto de Nutrición y Tecnología de Alimentos, Centro de Investigación Biomédica, Universidad de Granada, 18071 Granada, Spain; silviamolino@correo.ugr.es (S.M.); jarufian@ugr.es (J.Á.R.-H.)
[5] Instituto de Investigación Biosanitaria ibs. GRANADA, Universidad de Granada, 18071 Granada, Spain
* Correspondence: luisella.verotta@unimi.it; Tel.: +39-02-503-14114

Supplementary Materials

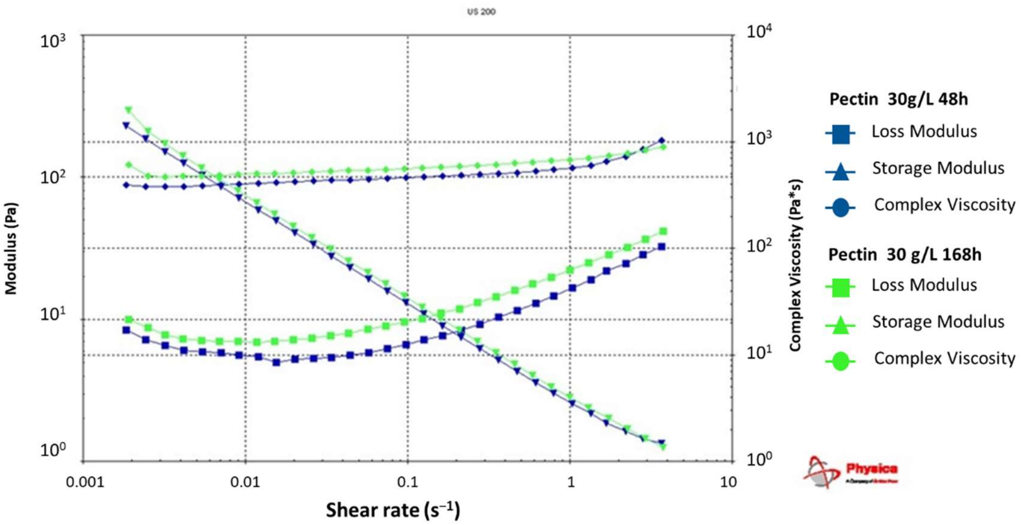

Figure 1. Rheological analysis of 30 g/L LM pectin gels after 48 h (green) and after 168 h (blue). Triangles: complex viscosity (η*); Circles: Storage Modulus (G'), Squares: Loss Modulus (G'') vs shear rate.

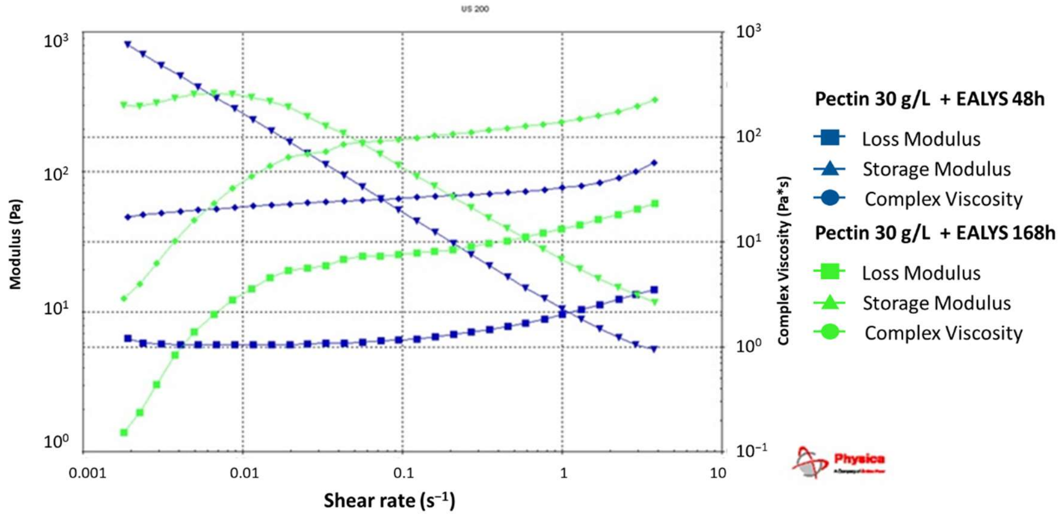

Figure 2. Rheological analysis of 30 g/L LM pectin gels loaded with EALYS after 48 h (blue) and after 168 h (green). Triangles: complex viscosity (η*); Circles: Storage Modulus (G′), Squares: Loss Modulus (G″) vs shear rate.

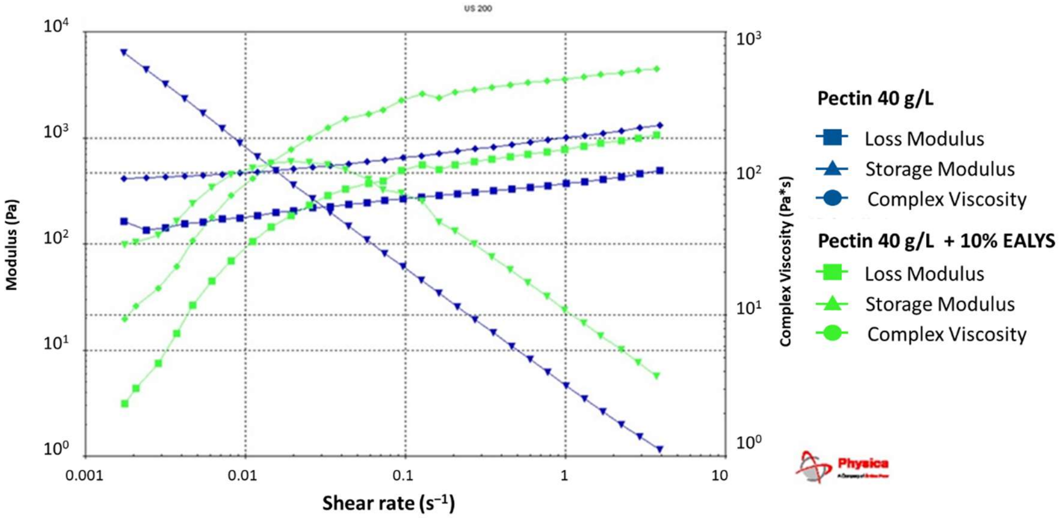

Figure 3. Rheological analysis of 40 g/L HM pectin gels (blue) and of 40g/L HM pectin gels loaded with EALYS (green). Triangles: complex viscosity (η*); Circles: Storage Modulus (G′), Squares: Loss Modulus (G″) vs shear rate.
